# Supplementary material for: Are carbon emissions trading and green financial instruments synergistic? -Comprehensive quantitative research based on content analysis
Source: PLoS One. 2024 Mar 7;19(3):e0298601. doi: 10.1371/journal.pone.0298601 (PMC10919601; doi:10.1371/journal.pone.0298601)
Supplement: S1 Data — (ZIP) [file pone.0298601.s001.zip › Data/地级市Z_F工作报告.zip!/╕╩╦α/╠∞╦«╩╨/└┤╫╘╣1⁄2╓┌║┼í╛┬φ┐╦╩2╛▌═°í┐_2017.docx]

各位代表:

现在,我代表市人民政府向大会作工作报告,请予审议,并请各位政协委员和列席人员提出意见。

一、市六届政府工作回顾

市六届人大一次会议以来的五年,是天水发展历程中很不平凡的五年。五年来,在省委、省政府和市委的正确领导下,在市人大、市政协的监督支持下,在社会各界的通力协作下,市政府坚持“主动作为、稳中求进、赶超进位、务求实效”的工作总基调,团结带领全市干部群众,攻坚克难,开拓进取,较好地完成了市六届人大一次会议以来确定的目标任务,实现了“十三五”发展良好开局,朝着全面建成小康社会、建设幸福美好新天水的目标迈出了更加坚实的步伐。

这五年,我们坚持以主动作为促赶超进位升级,是综合实力大幅提升的五年。全市大部分经济指标增速高于全省平均水平,主要经济指标增幅在全省排名逐年前移。预计2016年,全市实现生产总值601.7亿元,经济总量由2011年的全省第5位上升到去年的第3位;年均增长10.3%,增速由2011年的全省第13位上升到去年的第2位。完成固定资产投资664亿元,是2011年的2.3倍,年均增长17.85%,增速由2011年的全省第7位上升到去年的第6位。社会消费品零售总额288.1亿元,是2011年的1.9倍,年均增长13.98%,增速由2011年的全省第5位上升到去年的第1位。大口径财政收入121.49亿元,是2011年的2.02倍,年均增长15.09%,其中一般公共预算收入41.29亿元,是2011年的2.22倍,年均增长17.25%,增速由2011年的全省第11位上升到去年的第3位。一般公共预算支出242.96亿元,是2011年的1.83倍,年均增长12.83%,增速由2011年的全省第7位上升到去年的第1位。城镇居民人均可支配收入22682元,是2011年的1.73倍,年均增长11.7%,增速由2011年的全省第12位上升到去年的第6位。农村居民人均可支配收入6726元,是2011年的2.04倍,年均增长15.3%,增速由2011年的全省第4位上升到去年的第2位。金融机构存款余额1139.4亿元,是2011年的2.1倍,贷款余额734.18亿元,是2011年的2.7倍,增速均位居全省前列。直接融资58.94亿元,是2011年的15倍。

这五年,我们坚持以结构调整促提质扩量增效,是特色产业快速发展的五年。一是农村经济全面发展。“48113”现代农业推进计划深入实施,预计2016年果品、蔬菜、畜牧业总产值和劳务收入分别达到90亿元、68亿元、71亿元和120亿元,是2011年的2.58倍、2倍、2.94倍和1.9倍,农业四大主导产业收入占农民收入比重超过80%。八个现代农业示范园区和100个科学发展示范点累计完成投资46.2亿元,新培育农业龙头企业295家,新注册农民专业合作社3422家、家庭农场461家,武山县、甘谷县分别创建为国家级蔬菜标准化示范县和农业综合标准化示范县,“花牛苹果”荣获“中国驰名商标”。累计建成农村公路7141公里,完成生态造林63.55万亩,新修梯田163.39万亩,建设堤防510.2公里,解决了176.05万农村人口饮水安全问题。二是工业经济质效同步提升。预计2016年实现规模以上工业增加值118亿元,年均增长12.5%。五年实施千万元以上工业项目547项,完成工业固定资产投资654亿元,华天科技、长城电工、星火机床三大产业园一期相继建成,天水卷烟厂易地技改、太极集团羲皇阿胶生产线、西电高压电工触头生产基地、国家高低压电器质量监督检验中心、上海莺伦电动车武山生产线、三峡新能源张家川一期49.5兆瓦风电等项目建成投产。经济技术开发区扩区增容步伐加快,铁路电缆、长控公司等企业出城入园有序推进。众兴菌业、成纪药业成功上市,风动机械在“新三板”上市挂牌,108户企业在区域性股权市场挂牌。我市被确定为“宽带中国”示范城市、国家电信普遍服务试点城市,命名为“全国十大质量魅力城市”,获批创建“全国质量强市示范城市”,有效商标总量达到2381件,中国驰名商标达到16件,甘肃省著名商标116件,新增甘肃名牌产品56个、总数达到77个,名牌产品新增数和保有量居全省前列。三是第三产业发展步伐加快。预计2016年三产增加值达到297亿元,年均增长20%。商贸物流基础设施不断改善,天水商贸城、甘谷浙江商贸城、秦安中国西部小商品城、武山中乾物流园、天水肉类蔬菜流通追溯体系等项目加快实施,天水国际陆港建设有序推进,铁路物流中心可研通过评审,一批省外果蔬直营店建成运营,天水众创空间暨电子商务中心投入运行,武山县被列为国家级电子商务进农村综合示范县。非公有制经济健康发展,占生产总值比重由2011年的41%提高到51%。四是文化旅游融合发展。麦积山石窟列入世界文化遗产名录,伏羲庙等7个景区晋升为国家4A级景区,武山县被列入首批国家全域旅游示范区创建县,我市牵头的陇东南五市旅游联盟成立,麦积翠湖公园、金龙山文化旅游园、甘谷大像山文化主题公园、张家川回乡风情园、武山宁远生态园、清水轩辕文化产业园一期、麦积山温泉度假酒店等项目建成运营,麦积山石窟研究所科研基地、黄河雕漆文化产业园、佳·水岸小镇、台湾士林不夜城、峡门风情小镇、天水华昌欢乐城、娘娘坝丝路风情小镇、街亭古镇保护开发等项目顺利推进,天水火车站客车整备所建成投入使用,天水至兰州“麦积山号”、天水至西安“天水号”、天水至敦煌“伏羲号”等4列旅游列车开通,文化产业增加值年均增长34.48%,旅游综合收入年均增长28.2%。

这五年,我们坚持以投资拉动促基础条件改善,是城乡面貌显著变化的五年。全力实施“3341”项目工程,累计实施上亿元项目724个,总投资6915.4亿元。加强交通水利能源项目建设,天平铁路、十天高速、宝天高速过境段及街亭出口、庄天二级公路、洛礼二级公路、张家川县城至恭门火车站二级公路、凯迪生物质能源发电、中卫至贵阳天然气管道天水段、750千伏输变电、社棠330千伏输变电等重大项目全面建成,城区应急供水工程通水运营,宝兰客专天水段、国道310秦州至武山段升级改造、甘谷至麦积二级公路、城区引洮供水等项目加快建设。全市公路通车总里程突破1万公里,市县之间实现二级以上公路连接,4个县区通高速公路,所有乡镇和建制村通沥青(水泥)路。加强城市基础设施建设,五年共实施城建项目260项,总投资385.7亿元,已建成168个,完成投资276亿元。累计建成城市主次干道26条,建设桥梁24座,新建改建广场4个,硬化亮化小巷道280条,新建改建公厕104座,新增绿地500万平方米,赤峪路改造、藉河南路西延段、成纪大道、马跑泉大道东延段、藉河孙家坪大桥、藉河山水一号大桥、藉河双桥大桥、渭河峡口大桥、渭河社棠大桥、渭河甘谷杨赵大桥、渭河武山西关大桥、秦安宋场葫芦河大桥、天水建筑科技中心、成纪新城输配水管网、马跑泉公园提升改造、麦积区区府路西延段、西十里藉河生态公园等重点城建项目相继建成,秦州大道、麦积山大道、东柯大道、成纪新城路网一期、藉河罗家沟大桥、固体垃圾处理循环产业园、麦积污水管网改造、天水高铁南站综合交通枢纽及站前广场、市体育中心、秦州热源厂、成纪新城地下净水厂、有轨电车一号线、藉河红桥、甘肃建投天水总部经济城、藉河风情线一期延伸等项目加快建设,城区供热管网改造、秦州至三阳川隧道、藉河二十里铺大桥等项目前期工作有序推进,全国“智慧城市”试点全面启动,我市荣获“中国十大最美城镇”称号。预计2016年底,城市建成区面积增加到68平方公里、建设范围扩大到380平方公里、开发建设用地面积达到148平方公里,常住人口城镇化率45.2%,较2011年提高12个百分点。深入推进新农村建设和城乡一体化试点,全市12%的村建成省、市级新农村和美丽乡村示范村,39%的村建成整洁村。

这五年,我们坚持以深化改革促体制机制创新,是发展活力不断增强的五年。政府机构改革全面完成,事业单位改革完成分类工作,工商质监、食品药品、卫生系统、文化广电等管理体制调整理顺。公务用车制度改革有序推进,已完成市直部门车辆封存。农村改革稳步推进,甘谷县全国农村供销合作社综合改革试点工作取得明显成效,农村土地承包经营权确权颁证和不动产统一登记工作进展顺利,全市土地流转面积达到105万亩、流转率19%。完成撤乡改镇54个,建制镇占乡镇总数的89.4%。国资国企改革持续深化,营业税改征增值税顺利推行,“五证合一、一照一码”开始实行。市场主体达到11.7万户,是2011年的1.71倍,年均增长11.33%。交通银行、甘肃银行、兰州银行、浙商银行、中信建投证券、西部证券、平安养老、新华保险等15家金融机构入驻天水或设立办事机构。项目投融资方式不断创新,市级投融资平台累计融资103.26亿元,争取国开行、农发行等政策性银行贷款658.2亿元,通过“PPP”、“EPC”等方式实施项目77个、总投资590.53亿元。招商引资成效明显,累计签约招商项目1126项,新建续建项目到位资金预计达到1561.6亿元,年均增长40.5%。对外交流合作不断加强,关中—天水经济区发展规划深入实施,天水机场开通天水至重庆、天津、大连等地航班,天水至西安形成每日三班“航空快线”,2016年旅客吞吐量预计突破10万人次,是2011年的20.5倍。公祭伏羲大典、环青海湖自行车赛天水赛段、全国山地自行车冠军赛、全国少儿乒乓球比赛、中国龙舟公开赛、麦积山全国山地自行车邀请赛、国际传统射箭锦标赛、阿拉伯国家驻华使节天水行、影像丝绸之路·天水国际摄影双年展等活动成功举办,“中国绿色催化专家智库”组建成立,“麦积山论坛”永久性会址一期工程建成投入使用,2016绿色及可持续发展麦积山论坛成功举办。外贸进出口稳中向好,成立了天水海关办事处,预计2016年实现外贸进出口总额25亿元,是2011年的1.48倍。

这五年,我们坚持以重视民生促全面小康建设,是人民生活持续改善的五年。精准脱贫攻坚扎实推进,整合投入各类资金223.3亿元,在全省率先探索推行“双助贷”工程、“三户一孤”人员大病救助保险全覆盖项目、“三社合一”改革和“农户贷款、带资入股、就业分红”金融扶贫等模式,五年累计减贫101.37万人,贫困人口将由2011年底的133.15万减少到31.78万,贫困发生率由43.26%降低到10.33%。创新驱动战略深入实施,组织实施市级以上科技项目872项,取得科技创新成果706项,专利授权量2437件。教育事业全面发展,五年共投入教育经费203.2亿元,增幅达108.5%,实施教育项目3532个,建设校舍110.32万平方米,学校D级危房全面消除,建成行政村幼儿园538所,市一中麦积校区、市三中迁建、市一幼改扩建等项目加快建设,秦安县建设陇城教育园区的做法得到国家和省上的充分肯定。医疗卫生基础条件持续改善,建成标准化村卫生室1366个、乡镇卫生院业务用房和职工周转房宿舍95个,市妇幼保健院和秦州区人民医院迁建、市全科医师临床培训基地和儿童病区综合楼、市三院重症精神病住院楼、407医院内科楼全面完工,麦积区中医院和市二院实现合并,新农合参合率达到98.33%。公共文化体育设施不断完善,建成“乡村舞台”2491个、乡镇综合文化站113个、城市影院7座、农家书屋2557个、“历史再现”博物馆63个、“文化集市”7个,建成乡镇及社区体育惠民工程41个、行政村农民健身工程1031个。《天水通史》、《魅力天水》出版发行,《天水大辞典》通过终审,创排了《一画开天》、《大秦文公》、《麦积圣歌》、《轩辕大帝》、《陇上铁汉》、《邓宝珊将军》、《姜维》、《关山月》等精品剧目,拍摄了《苦乐村官》、《幸福快车》等影视剧,大型纪录片《麦积山石窟》、《祖脉天水》在央视播出。城镇就业总量持续增加,累计新增城镇就业33.62万人。社会保障体系日益健全,退休职工养老金平均待遇、城镇居民医保报销比例逐年提高,城市低保、农村低保、五保供养标准分别比2011年提高85.28%、160.49%和104.02%。累计建设保障性住房7.22万套556.87万平方米,实施农村危房改造8.35万户,发放廉租住房补贴9.77万户2.42亿元。特大暴洪和群发性地质灾害恢复重建基本完成。成功创建全国双拥模范城“五连冠”。生态建设不断加强,城区治污成效明显,创建国家级生态乡镇和生态村各3个,全市森林覆盖率达到33.21%,环保意识普遍提升,“天水蓝”成为靓丽城市名片。安全生产形势稳定,民族宗教事务和顺,“平安天水”建设深入推进,社会大局和谐稳定。统计调查、档案史志、防震减灾、人民防空、社会团体、妇女儿童、老龄老干、老年教育、残疾人事业和关心下一代等工作都取得了新的成绩。

这五年,我们坚持以转变职能促行政效能提升,是政府建设全面加强的五年。全面落实省委、省政府和市委各项决策部署,自觉依法接受市人大及其常委会的法律监督和工作监督、市政协及参加单位的民主监督、各界各方的群众监督和舆论监督,全面执行人大决议,充分吸纳政协意见,五年办结人大代表建议1013件、政协提案1200件。严格执行中央“八项规定”、国务院“约法三章”、省委“双十条”规定,认真落实政府系统党风廉政建设主体责任,巩固党的群众路线教育实践活动成果,扎实开展“三严三实”专题教育和“两学一做”学习教育,持续推进“效能风暴”行动,有效整改“四风”和“不严不实”等问题,政府自身建设得到加强。坚持依法行政,“六五”普法全面完成,政府法治意识和能力进一步增强。不断深化行政审批制度改革,深入推进政务公开,大力发展电子政务,五年累计取消调整下放行政审批事项350项,市、县区政府部门“三张清单一张网”建成并上网运行,市公共资源交易中心逐步完善运行,行政效能不断提升。

各位代表,过去的五年,是我市综合实力显著提升、城乡面貌显著变化、人民生活显著改善的五年。这些成绩的取得,是省委、省政府正确领导、亲切关怀的结果,是市委统揽全局、科学决策的结果,是市人大及其常委会、市政协、各民主党派、工商联及社会各界有效监督、鼎力支持的结果,是全市广大干部群众及各族各界人士和衷共济、团结奋斗的结果。在此,我代表市人民政府,向奋战在全市各条战线上的广大干部群众,向各民主党派、人民团体、工商联和无党派人士,向离退休老同志,向关心家乡的天水籍和天水工作过的在外人士,向驻市人民解放军、武警消防官兵和驻市单位,表示崇高的敬意和诚挚的感谢!

各位代表,五年的砥砺奋进,取得的成绩令人振奋;五年的探索实践,积累的经验弥足珍贵。我们深切体会到,搞好政府工作应当进一步把握以下几点:必须坚持把解放思想作为永恒主题,吃透市情,把握上情,坚定不移地、创造性地贯彻落实省委、省政府和市委的决策部署;必须坚持把加快发展作为第一要务,始终保持赶超进位、争创一流的精神状态,坚持稳中求进、好中求快,把天水的发展放在全国全省大局中去谋划去推进;必须坚持把发挥优势作为着力重点,始终从天水实际出发,充分发挥自然生态良好、文化底蕴深厚、交通区位便利、旅游资源富集等比较优势,走具有天水特色的科学发展之路;必须坚持把项目建设作为重要支撑,充分发挥投资对经济社会发展的拉动作用,以大项目带动大投资、促进大发展,做到大项目顶天立地、好项目真正落地;必须坚持把改革开放作为动力源泉,在精准脱贫攻坚、重大项目实施、城市开发建设、投资融资保障等方面探索创新,不断深化重点领域改革,全方位加强对外交流合作,最大限度激发内生动力和发展活力;必须坚持把以人为本作为根本要求,以富民为本、惠民为先、利民为重,把民生事业放在重中之重的位置去考虑,让改革发展的成果更多惠及城乡群众;必须坚持把务实苦干作为一贯作风,锲而不舍,驰而不息,牢记初衷使命,强化责任担当,提升工作效能,推动政府各项工作取得更大成效。

各位代表,在肯定成绩的同时,我们清醒地认识到,发展中还存在不少困难和问题:经济总量不大,自然资源禀赋较低,农业产业化水平不高,工业提质扩量难度较大,服务业发展相对滞后,赶超进位和加快发展的任务依然艰巨;产业结构不尽合理,战略性新兴产业比重偏低,非公经济发展比较缓慢,创新创业活力不足,调整结构和转变方式的任务依然艰巨;基础设施建设相对滞后,城市管理问题突出,生态环境保护和节能减排降耗的任务依然艰巨;农村贫困面大,贫困程度深,脱贫攻坚和全面小康建设的任务依然艰巨;社会保障体系还不完善,基本公共服务供给不够充足,社会管理水平还需提升,改善民生和维护稳定的任务依然艰巨;一些部门和公务人员工作作风不实、办事效率不高、执行力不强,慵懒散慢问题突出,提升工作效能和优化发展环境的任务依然艰巨。对这些问题,我们将以更大的决心、更大的力度、更有效的措施认真加以解决。

二、今后五年的发展目标和主要任务

今后五年,是我市全面建成小康社会的关键时期。我们要充分利用当前可以大有作为的重要战略机遇期,紧紧抓住国家深入实施“一带一路”战略、建设关中—天水经济区、推进精准脱贫攻坚和省上全力打造三大国家级战略平台等重大机遇,准确把握新常态下天水发展所处的坐标定位和阶段特征,有效应对改革发展中遇到的各种困难和挑战,奋力推动天水经济社会实现更快更好的发展。只要全市上下心往一处想、智往一处谋、劲往一处使,我们完全有基础、有条件、有信心、有能力,战胜各种困难,实现赶超进位,在新的历史征程中赢得主动、赢得优势、赢得未来。

按照市第七次党代会绘制的宏伟蓝图和总体部署,今后五年政府工作的指导思想是:全面贯彻落实中央各项战略决策、方针政策和习近平总书记系列重要讲话精神,按照省委、省政府和市委的工作部署,牢固树立创新、协调、绿色、开放、共享的发展理念,紧紧围绕全面建成小康社会总目标和“十三五”经济社会发展规划,继续坚持“主动作为、稳中求进、赶超进位、务求实效”的工作原则,着力保持合理增长,着力调整优化结构,着力提高发展质量,着力深化改革开放,着力推进脱贫攻坚,着力保障改善民生,着力维护和谐稳定,为加快建设区域中心城市和现代化大城市、丝绸之路经济带历史文化名城继续努力奋斗。

经济社会发展的预期目标是:坚持预期性目标与约束性指标相结合,经济发展与社会发展相统一,力争实现“一个赶超、三个控制、五个高于”。“一个赶超”即经济总量和综合实力实现赶超进位,在全省非省会城市中名列前茅;“三个控制”即居民消费价格指数、城镇登记失业率、环保和能耗重点指标控制在省上下达的指标以内;“五个高于”即生产总值、固定资产投资、财政收入、城镇居民人均可支配收入、农村居民人均可支配收入增幅高于全省平均水平。具体指标是,生产总值年均增长8%,固定资产投资年均增长10%,社会消费品零售总额年均增长10%,一般公共预算收入年均增长8%,城镇居民人均可支配收入年均增长9%,农村居民人均可支配收入年均增长12%。围绕实现以上目标,我们将重点做好以下六个方面的工作:

(一)把握精准性全力实施“一号工程”,坚决打赢精准脱贫攻坚决胜战。一是巩固提升“十个全覆盖”。即贫困村通沥青(水泥)路、自然村通动力电、乡镇中心幼儿园、标准化村卫生室、村便民超市、村文化图书室、村体育活动设施、乡村舞台、贫困村扶贫互助资金协会、1500人以上行政村幼儿园实现“全覆盖”。二是加快实现“四个村村通”。即村村通沥青(水泥)路、村村通安全饮水、村村通有线广播电视、村村通4G互联网络。三是全力抓好“六项重点工作”。即加大危房改造力度,全面消除农村贫困户和非贫困户D级危房;推进易地扶贫搬迁,搬迁贫困群众2.11万户9万人,实现具有搬迁条件和意愿的贫困户应搬尽搬;加快富民产业培育,扶持贫困户发展果品、蔬菜、畜牧、劳务等主导产业和中药材、马铃薯、花卉等特色优势产业,“十三五”末贫困村农民人均可支配收入达到10000元以上;加强项目资金整合,采取财政专项资金+整合项目资金+金融信贷资金+社会帮扶资金+群众自筹资金等模式,充分放大资金使用效益;全面落实“三户一孤”人员大病救助保险全覆盖,为建档立卡贫困户、农村低保户、农村五保户和城乡孤儿全部办理大病救助保险;全面推进“双助贷”工程,全市每个行政村扶贫互助资金总量平均达到100万元以上。四是确保实现“一个总目标”。加快落实“1+17”精准扶贫方案,严格执行“4342”脱贫验收责任体系,强化督查考核,狠抓措施落实,确保2020年前现行标准下农村贫困人口全部脱贫,贫困县区全部摘帽。

(二)践行新理念力促产业转型升级,努力推动经济发展再上新台阶。一是推进工业提质扩量工程。坚定不移实施“工业强市”战略,培育发展5户年销售收入上50亿元的产业化企业、10户年销售收入上10亿元的骨干型企业、100户以上年销售收入上亿元的成长性企业,发挥好8户省级和21户市级战略性新兴产业骨干企业的示范带动作用,全力实施长城电工电器产业园、华天科技产业园、工业博物馆、电工产品综合检测实验研究基地、岐黄中医药产业园等重点工业项目,推动机械制造、电工电器、电子信息、医药食品、建筑建材、能源化工等产业优化升级,打造年销售收入上百亿元的六大产业集群,逐步建设产值上千亿元的国家级开发区,成功创建“全国质量强市示范城市”。二是加快农业产业化进程。深入实施“48113”现代农业推进计划,稳定发展粮食生产,做大做强果品、蔬菜、畜牧、劳务“四大主导产业”,完善提升“八个现代农业示范园区”,持续推进“十个百万工程”,培育壮大“百强龙头企业”,着力打造“三条城乡一体化示范长廊”,每年办好“100个科学发展示范点”,“十三五”末,农业四大主导产业均达到“百亿元产业”,力争总产值超过600亿元。三是促进文化旅游提速发展。认真实施华夏文明传承创新区“136”工程和《天水市文化与旅游深度融合发展规划》,加快陇东南“祖脉”文化旅游圈建设,着力打造羲皇故里·世界华人寻根祭祖圣地、人文天水·丝绸之路历史文化名城、陇上江南·宜居宜业宜游生态家园三大品牌,积极推动关中—天水经济区、陇东南、陕甘川毗邻地区三大文化旅游联盟工作,加快推进麦积山、伏羲庙、大地湾三个大景区建设,重点实施天水华昌欢乐城、麦积全国综合养老示范基地及翠园文化产业园、台湾士林不夜城、天水大剧院、数字麦积山及游客中心、甘谷毛家坪遗址公园、秦安凤山文化生态园、清水轩辕文化产业园、张家川马家塬遗址公园、武山文化产业园等项目,“十三五”末,全市文化产业增加值达到40亿元,旅游综合收入达到280亿元。四是提升现代服务业发展水平。支持发展多层次多门类和跨境电子商务,创建“国家级电子商务示范城市”,全市电子商务交易额年均增长30%以上,网络零售额年均增长35%以上。积极发展科技、金融、商务等生产性服务业和健康、养老、旅游等生活性服务业,全市服务业增加值达到450亿元,年均增长9%。

(三)聚焦大项目持续增加有效投资,不断改善城乡发展基础条件。一是构建区域交通枢纽。加快实施“6873”交通突破行动,建成天水至平凉高速公路、国道310秦州至武山段升级改造、陕西陇县至甘谷至通渭榜罗公路、甘泉至麦积山景区、麦积区潘集寨至街亭出口至温泉、洛门至水帘洞旅游公路、武山至漳县公路等项目,创造条件实施静宁至天水高速、天水南绕城高速和秦安莲花至张家川至陕西陇县石槽沟公路等项目。全面完成农村公路通畅工程,改造提升县乡公路,逐年对具备条件的自然村道路进行硬化,“十三五”末实现“县县通高速、乡镇通国省道、村村通客车、打通出口路”的目标;建成宝兰客运专线,推进兰州至天水至汉中城际铁路、天水至陇南铁路项目;完成天水机场迁建。二是加强水利能源保障。加快建设城区引洮供水工程,尽早实施曲溪城乡供水、引洮二期配套工程、张家川东部供水、武山龙台水库、清水汤浴水库、麦积黄家峡水库等项目,配合做好白龙江引水项目前期工作,从根本上解决城乡供水问题;积极争取华电2×350兆瓦热电联产、清水2×1000兆瓦火电、甘谷金山光伏发电、甘谷古坡汇能风电等项目,全市所有县区实现天然气管道供应。三是打造物流信息中心。高标准建设天水国际陆港,完成天水商贸城、甘肃东部甘谷物流中心、秦安中国西部小商品城等项目,启动建设天水汽车博览城、天水粮食区域物流中心、武山5万吨冷链物流等项目。全力争取国家和省上布局设立互联网骨干直联点,规划建设大数据、“云计算”区域中心,完善地理信息大数据共享交换平台,努力打造数字天水。四是推动绿色低碳发展。实施渭河干流天水段及其支流、西汉水等重点区域生态综合治理项目,推进新一轮退耕还林、天然林保护二期、三北五期防护林、国家公益林管护等林业生态工程,打造国家生态安全屏障综合试验区“南部基地”;办好“中国绿色催化专家智库”和“绿色及可持续发展麦积山论坛”,着力打造国家级高端智库和具有国际影响力的绿色可持续发展论坛品牌。

(四)突出宜居性推动城市组团发展,加快建设区域中心城市和现代化大城市。一是优化城市发展布局。编制完成第四版城市总体规划,争取设立三阳川新区,逐步形成“五区三县”城市发展格局。按照“东西延伸、相向对接、南北优化、旧城更新”的总体思路,统筹抓好古城保护、旧城更新和新城开发,实施西关古城保护与复兴项目,启动天水郡、枣园村、东团庄、北园子、马跑泉、道北等片区和羲皇大道、藉河南路沿线更新改造,加快秦州新城、成纪新城、麦积新城、颖川新城、东柯新城、中梁空港城、三阳川新区、社棠工业城八大新城区建设,不断拓展城市发展空间。二是完善城市服务功能。加快建成秦州大道、麦积山大道、东柯大道、城市轨道交通、藉河罗家沟大桥、藉河二十里铺大桥、渭河麦积城区三号大桥、秦州至三阳川隧道等一批道路桥梁项目;加快并建成秦州热源厂和成纪新城、麦积新城、颖川新城、东柯新城热源厂及城区供热管网改造等一批供热项目;加快并建成成纪新城地下净水厂、固体垃圾处理循环产业园等一批垃圾污水处理项目;加快建设社区市场、公交车停靠点、公共自行车、公共停车场、小巷道整修硬化、人行道铺装改造、公厕改造提升等一批城建民生项目;加快推进“智慧城市”、“海绵城市”和城市地下综合管廊建设。加强城市综合管理,深入开展“三城联创”活动,下决心整治各种“城市病”,加快创建国家园林城市、国家卫生城市和中国最佳历史文化旅游城市。三是加快新型城镇化建设。重视各县县城建设,发展壮大县域经济,推动县区之间功能互补、协调发展。高起点建设省级重点镇麦积区甘泉民俗风情小镇,实施娘娘坝丝路风情小镇、孙集文化小镇、街亭古镇保护开发、百花小镇、峡门风情小镇、中梁空港小镇等项目,打造一批各具特色的风情小镇,力争全市常住人口城镇化率提高到53%以上。四是加大环境保护力度。持续推进藉河至渭河城区段生态环境综合治理、“两山两河”百公里生态长廊建设,打好大气、水、土壤污染防治三大战役,下决心取缔城区燃煤小锅炉,实施县区城镇污水处理厂提标改造和污泥集中处置工程,实现重点镇生活污水、垃圾处理设施全覆盖,加强河道治理和农村环境整治,建立土壤环境基础数据库,全面完成“十三五”污染减排目标任务。

(五)立足求实效深化重点领域改革,进一步激发发展活力和动力。一是着力推进各项改革。深入推进供给侧结构性改革,认真落实“三去一降一补”任务,不断优化供需结构,提升供给质量和效率。深化行政审批制度改革,加大简政放权力度,加强“三张清单一张网”动态管理,全面推行“网上行权”,提高审批效率和服务水平。启动国有林场改革,推进农村供销合作社全面改革,完成农村土地承包经营权确权登记颁证。深入推进“先照后证”改革,全面推行“五证合一、一照一码”登记制度。深化国资国企改革,支持骨干企业通过转让经营权、出让股权、资产重组等方式发展混合所有制经济。二是创新投资融资机制。提升市级投融资平台运作水平,推广运用“PPP”等项目建设运营模式,培育发展各类投资基金。加快多层次资本市场发展,强化金融监管和风险防范,推动5户企业直接上市融资,培育200户以上企业在全国性及区域性股权市场挂牌托管,直接融资规模突破100亿元。三是不断扩大对外开放。加快关中—天水经济区建设,提升公祭伏羲大典层次和水平,办好重大体育赛事活动,多方位提升天水知名度和影响力。借助丝绸之路(敦煌)国际文博会、兰洽会等重大节会活动,加强天水特色产品和优势资源宣传推介,引进实施一批规模大、效益好、高科技、无污染的招商项目。

(六)着眼惠民生不断增进人民福祉,努力创造富裕文明幸福的美好生活。一是协调发展社会事业。坚持教育优先发展战略,完成义务教育“全面改薄”任务,加快发展学前教育,实施职业教育“22651”计划,普及高中阶段教育,重视加强特殊教育和老年教育,整合天水农校、天水卫校、天水职校等职业教育资源,提高办学职级和办学水平,积极创办天水大学。加快创新型城市建设,完善城乡科普基础设施,激发大众创业、万众创新活力。深化医药卫生体制改革,推动医疗卫生资源向基层倾斜,努力建设陇东南国家中医药养生保健旅游创新区。加快文化体育事业发展,建成市体育中心、市文化馆、市博物馆、市图书馆、市文广传媒中心等项目。二是重视做好就业保障。统筹推进高校毕业生等重点群体就业工作,开展贫困家庭子女、未升学初高中毕业生、农民工、失业人员和转岗职工、退役军人免费接受职业培训行动,做好公益性岗位托底安置工作。“十三五”全市年均新增城镇就业6万人以上,城镇登记失业率控制在4%以内。实施全民参保计划,落实机关事业单位职工养老保险和城乡居民大病保险制度,做好被征地农民养老保险工作,大力发展职业年金、企业年金和商业养老保险,城乡居民社会养老保险覆盖率达到95%以上,城镇职工基本医疗保险、城镇居民基本医疗保险、生育保险、工伤保险动态覆盖率达到98%以上。三是维护社会和谐稳定。不断创新社会治理,组织实施“七五”普法规划,推进“平安天水”、“法治天水”建设,完善立体化社会治安防控体系,加强社会稳定风险评估,严厉打击违法犯罪活动,有效化解各类社会矛盾。加强防灾减灾和人防工作,坚持不懈抓好安全生产,深入实施食品药品安全战略,及时防范和处置突发问题。深化双拥共建活动,推动军民融合发展,实现争创全国双拥模范城“六连冠”目标。着力提升市民文明素质和社会文明程度,努力争创“全国文明城市”。加快“两个共同”示范县建设,依法管理宗教事务,促进民族地区经济社会发展。高度重视人才队伍建设,进一步完善人才引进、使用和评价机制。重视加强统计调查、档案史志、社会团体、妇女儿童、老龄老干、老年教育、残疾人事业和关心下一代等工作。

三、2017年政府重点工作

2017年,是新一届政府的开局之年,也是实施“十三五”规划的重要一年。全市经济社会发展的主要预期目标是:生产总值增长8%,规模以上工业增加值增长9.5%,固定资产投资增长10%,社会消费品零售总额增长9%,一般公共预算收入增长8%,城镇居民人均可支配收入增长9%,农村居民人均可支配收入增长12%,居民消费价格指数涨幅控制在3%以内,环境保护和单位生产总值能耗完成省上下达指标。

(一)坚持在富民增收中加快农业农村发展。一是精准聚焦施策抓脱贫。完善精准扶贫大数据平台,继续抓好产业培育、群众增收、易地搬迁、危房改造、学前教育、电子商务、农村养老、物流快递、科技服务、技能培训等重点工作,市、县区财政专项扶贫资金突破6亿元,整合扶贫资金48亿元,搬迁贫困户4622户21314人,完成贫困户危房改造1.2万户,选聘建档立卡贫困人口生态护林员1241名,年内减贫16万人,甘谷县、武山县、麦积区实现整体脱贫。二是发展富民产业促增收。持续打造果品、蔬菜、畜牧、劳务“四大百亿元主导产业”,新建优质果品基地10万亩,新增蔬菜播种面积2万亩,新建和改扩建标准化规模养殖场80个,有组织输转劳动力70万人,果品、蔬菜、畜牧三大产业产值分别达到100亿元、80亿元和77亿元,劳务收入达到132亿元。加快马铃薯、中药材、花卉等特色产业发展,新建中药材基地5万亩。三是改善基本条件强基础。全面落实科技增粮措施,粮食播种面积稳定在460万亩左右,确保正常年景粮食总产达到120万吨以上。完成造林20万亩,兴修梯田12万亩,新增有效灌溉面积1万亩,修建堤防工程60公里,治理水土流失面积250平方公里。

(二)坚持在提质扩量中推动工业转型升级。一是立足“扶优”做大骨干企业。全力打好战略性新兴产业培育攻坚战,加大对8户省级骨干企业和21户市列骨干企业扶持力度。抢抓国家支持贫困地区企业挂牌上市优惠政策,推动华洋电子、华圆科技、海林中科、锻压机床等企业上市或在“新三板”挂牌。二是围绕“扩量”抓好工业项目。加快长城电工电器产业园、华天科技产业园二期、工业博物馆、电工产品综合检测实验研究基地、长城果汁10万吨果蔬汁罐装示范基地、西北永新管材基地、岐黄中医药产业园、天河酒业生态酿酒城技改扩建、张家川三峡新能源风电、天森药业产业基地等工业项目建设,盯紧落实天水烟用包装印刷材料、大型客机改货机、高端无人机、复合电致发光材料及器件技术产业化(OIEL)、石墨烯、锂资源综合应用、陶瓷膜技术等有一定基础的项目,积极引进实施一批新的工业项目。三是紧盯“增容”推进开发区建设。加快冀城产业园规划建设,完善社棠工业园服务功能,重视园区招商引资,力争开发区工业增加值增长12%。加快岐黄药业、长控公司、天宝塑业、铁路电缆、海辰轴承等企业出城入园工作。高度重视县区工业园区和工业集中区建设,推动县域工业经济发展。四是突出“提质”加快企业技术创新。积极创建“全国质量强市示范城市”,全面实施电信普遍服务试点建设项目,争取建设大数据、云计算区域中心,加快建设国家电线电缆质量监督检验中心,年内争创省级以上企业技术中心或工程中心3家、省级以上技术创新示范企业2家、中国驰名商标1件、甘肃名牌产品8个。

(三)坚持在融合创新中提升三产发展水平。一是完善商贸基础设施。加快天水商贸城建设并投入运营,启动建设天水大剧院、成纪大酒店,加快推进天水国际陆港、武山中乾物流园、秦安中国西部小商品城、甘谷汽车城、天水城市蔬菜仓储分拣中心、瀛池蔬菜批发市场迁建等项目,改造提升乡镇农贸市场和社区便民市场。二是加快文化旅游发展。编制《陇东南祖脉文化旅游圈天水核心区建设规划》,推进大地湾国家考古遗址公园保护建设,加快实施天水华昌欢乐城、数字麦积山及游客服务中心、翠园文化产业园、娘娘坝丝路风情小镇、佳·水岸小镇、峡门风情小镇、百花小镇、街亭古镇保护开发、秦州印象等项目,创排《天水天秀》等剧目,推动伏羲庙争创国家5A级旅游景区,支持武山县、麦积区、秦州区创建国家和省级“全域旅游示范区”,全年旅游接待人数、综合收入分别增长22%和23%。三是促进消费转型升级。加快建设淘宝网“特色中国·天水馆”,实施电子商务进农村综合示范项目、电商扶贫试点和“快递下乡”工程,培育发展天水电商中心、天水美通进出口公司、天水农昕花牛电子商务公司等一批电商示范企业。加快实施台湾士林不夜城、居然之家、秦州景园城、福田城市综合体、天庆城市综合体等重点项目,全力打造城市商业聚集区。四是提高对外贸易水平。积极申报设立天水海关,推进天水保税物流中心、天水铁路口岸设立工作。落实我市与中东贸易框架协议,建立与迪拜纳世利中东贸易对接机制,扩大农产品、特色工艺品、电子电器等产品在中东、中亚等市场的出口份额,外贸进出口总额增长8%以上。

(四)坚持在完善功能中加强城乡基础建设。一是重视城市建设管理。全面建成商贸城路网、成纪广场、藉河生态环境综合治理一期续建、城区引洮供水、秦安县解放路改造、张家川县迎宾大道等项目,加快建设有轨电车一号线、成纪新城路网、麦积新城路网、秦州热源厂、成纪新城地下净水厂、固体垃圾处理循环产业园、藉河罗家沟大桥、市体育中心、武山县体育馆等项目,实施和争取实施秦州至三阳川隧道、岷山厂片区路网、藉河南路西延段二期工程、藉河二十里铺大桥、渭河麦积城区三号大桥、藉河生态环境综合治理二期、曲溪城乡供水工程管网、甘谷大像山公园、渭河武山城区段综合治理、清水轩辕湖二期等项目,推进“智慧城市”、“海绵城市”建设,在新区开发中探索开展城市地下综合管廊建设,积极推行“市民卡”一卡通工作。更加重视城市治理,加强城市食品、蔬菜、餐饮市场规划建设及归店管理,集中力量整治乱建房、乱停车、乱采石、乱采砂、乱摆摊点、乱堆乱放、乱倒垃圾、乱垦植被和环境卫生脏乱差等“八乱一差”问题。二是提升交通通达能力。年内建成310国道秦州至武山段、秦州大道、麦积山大道、东柯大道、甘泉至麦积山景区旅游公路、洛门至水帘洞旅游公路、高铁天水南站综合交通枢纽及站前广场、高铁麦积东岔汽车站、天定高速藉口出口、天巉公路“三站合一”三阳川收费站、天水汽车西站、麦积客运站、甘谷客运中心等项目,加快天平高速公路、陇县至榜罗公路秦安至甘谷礼辛段、礼辛至谢家湾公路、清水至社棠公路、清水汽车站等项目建设进度,启动实施陇县至榜罗公路清水至秦安段、长宁至清水段,推进静宁至天水高速天水段、天水南绕城高速公路等项目前期工作。三是加强生态环境治理。实施秦州北山植物园、吕二沟森林公园等项目,完善马跑泉公园、人民公园、诸葛军垒公园、儿童乐园基础配套设施,全面开展城区河道环境综合整治、居住小区(庭院)绿化提升改造和拆墙透绿工程,力争成功创建国家园林城市。加快城区集中供热、燃煤锅炉改造、煤炭市场建设,建成县级环境空气质量监测点,抓好建筑施工扬尘管理、煤质监管、渣土车管理、烧烤及饮食摊点归店经营、黄标车淘汰、禁燃区管控等工作,深入开展渭河城区段、葫芦河水污染防治,推动环境质量持续改善。

(五)坚持在改革开放中增强发展内生动力。一是抓好重点改革。全面完成农村土地承包经营权确权登记颁证工作,依法推进农村土地承包经营权有序流转。抓好精准脱贫农民合作综合服务社建设、“三户一孤”人员大病救助保险、“双助贷”工程等工作。积极推进秦州合行、麦积合行改制组建为天水农村商业银行。完成行政单位车改工作。全面推行“五证合一、一照一码”登记制度,加大非公经济和中小微企业扶持力度,非公经济增加值增长11%以上。二是重视招商引资。围绕传统优势产业和战略性新兴产业,发挥企业主体和园区平台作用,主动承接产业转移,及时跟进落实签约项目,招商引资到位资金增长12%以上。三是加强交流合作。深入推进关中—天水经济区建设,加强与丝绸之路经济带沿线城市的交流合作,办好公祭伏羲大典、中国龙舟公开赛、麦积山全国山地自行车邀请赛、国际传统射箭锦标赛等文化体育活动,开通天水至重庆直达航线和天水至兰州及省内部分城市航线。依托“中国绿色催化专家智库”和“绿色及可持续发展麦积山论坛”,推动更多绿色低碳产业化项目在我市落地实施。

(六)坚持在统筹协调中发展各项社会事业。一是推动教育稳步发展。继续实施市区教育资源提升“2521”计划,加快市一中麦积校区、市三中迁建、全面改薄、贫困村幼儿园等重点项目建设,确保市一中麦积校区秋季顺利招生。全力推动义务教育均衡发展,力争秦州、麦积、甘谷三县区顺利通过义务教育发展基本均衡县评估验收。实施职业教育发展“22651”计划,积极推动职业教育学校资源整合、改革升级工作。二是加大科技创新力度。争取国列、省列科技项目30项以上,实施市列科技项目100项。加快“大型电气传动系统与装备技术”国家重点实验室建设,抓好天水众创空间暨电子商务中心等省级众创空间、省级电子商务示范基地建设与运营。三是完善文化服务体系。加快“乡村舞台”和城镇社区综合文化场所建设,推进“历史再现”工程和遗址公园保护建设,新建工业博物馆、地质博物馆、广电博物馆等各类博物馆10个,加快市文化馆、博物馆、图书馆新馆建设,做好传媒中心等项目基础工作。实施广电网络全覆盖工程,完成34个乡镇转播台站无线数字化补点建设任务。四是提升医疗卫生水平。积极推进分级诊疗和医师多点执业,推行新农合、民政救助一站式服务,实现新农合“一卡通”使用全覆盖。加快市残疾人康复中心和乡镇中心卫生院、村卫生室标准化建设,扎实推进麦积全国综合养老示范基地、国家中医药养生保健旅游创新区建设。

(七)坚持在服务大局中维护社会和谐稳定。一是促进城乡创业就业。实施“高校毕业生就业促进计划”和“大学生创业引领计划”,引导扶持2000名高校毕业生到企业服务或创办企业,为创业人员提供创业担保贴息贷款3亿元,应届高校毕业生就业率达到85%以上,新增城镇就业6万人,城镇登记失业率控制在4%以内。二是加强社会保障工作。统筹做好全民参保、机关事业单位职工养老保险和城乡居民大病保险等工作,提高城乡低保标准和特困人员救助供养补助水平。完善企业及机关事业单位工资正常增长机制,提高离退休人员基本养老金水平。加强保障性安居工程建设,有效解决城镇低收入家庭和新就业人员、外来务工人员住房困难问题。三是提升社会治理水平。严格落实“党政同责、一岗双责、齐抓共管、失职追责”的安全生产工作机制,强化企业主体责任,加强重点行业和领域隐患排查整治,严防各类生产安全事故。深入推进信访制度改革,及时办理答复群众通过“市委书记市长留言板”、“市长信箱”等渠道反映的问题。深入开展矛盾纠纷排查调处,加强防暴处突和反恐维稳工作,依法严厉打击各类违法犯罪活动,努力营造和谐稳定的社会环境。

(八)坚持在推动发展中倾力办好民生实事。在全面完成省列民生实事的同时,市政府将集中力量办好市列民生实事:(1)在继续实施好市区教育资源提升“2521”计划的基础上,年内在市区新建、迁建、扩建初中2所、小学6所、幼儿园10所,下决心解决城区学生“入学难”、“入园难”问题。(2)在市区人流密集交通路口建设过街天桥4座,缓解交通压力,方便群众出行。(3)在市区新建停车场10处,新增停车位5000个以上,逐步缓解城区“停车难”问题。(4)为全市精准扶贫建档立卡贫困户、农村低保户、农村五保户和城乡孤儿等“三户一孤”人员全部办理大病救助保险,着力解决贫困户因病致贫的问题。(5)年内实现贫困村快递服务网点、电子商务站点、4G网络“全覆盖”。(6)全市完成100条小巷道综合整治,完成10万平方米人行道透水砖透水混凝土铺装改造,在市区改造提升公交站点200个,新建小花坛100个,新增绿地面积6000平方米。(7)实施农村危房改造1.2万户,全面消除农村D级危房。(8)重视解决城镇低收入群体住房困难问题,实施棚户区改造1.5万套。(9)在去年列支2亿元的基础上,市财政今年预算安排2亿元,为秦州、麦积两区被征地农民养老保险提供补贴。(10)全市完成易地扶贫搬迁7667户35124人,其中搬迁建档立卡贫困户4622户21314人。(11)对城区150座1000蒸吨燃煤锅炉进行提标改造或天然气改造,实现城区燃煤锅炉全部改造,让“天水蓝”更蓝。(12)新建乡镇及社区体育健身中心6个、行政村农民体育健身工程60个。

四、全面加强政府自身建设

实现今后五年的目标,全面建成小康社会,对政府工作提出了新的更高的要求。我们必须坚持不懈加强政府自身建设,进一步改革创新,扎实工作,努力做到为人民服务、对人民负责、受人民监督、让人民满意。

(一)坚持依法行政,着力建设法治政府。坚定服从市委的领导,自觉接受市人大及其常委会、市政协和社会各界的监督,认真落实市人大及其常委会的决议决定,认真吸收市政协及社会各界的意见建议,改进人大代表建议和政协提案办理方式。全面贯彻《法治政府建设实施纲要》,加强市政府立法工作,及时向市人大及其常委会提出年度和五年立法规划建议。坚持公众参与、专家论证、风险评估、合法性审查和集体讨论决定,推进政府决策民主化、科学化和法治化。扎实有效推进政务公开,完善政府新闻发布制度,保障群众的知情权、参与权和监督权。

(二)加快职能转变,着力建设效能政府。加大简政放权力度,深化行政审批制度改革,做好行政审批事项的“接、放、管、服”,规范市、县区政府“三张清单一张网”管理,加快电子政务建设,完善各级政务大厅职能,拓展网上并联审批平台,切实提高行政审批效率。进一步精简会议文件,不断改进会风文风,腾出更多时间和精力抓工作、促落实。持续推进“效能风暴”行动,严格目标责任考核,强化行政效能问责,从严查处庸政、怠政、懒政和为官不为等现象。

(三)改进工作作风,着力建设服务政府。牢固树立宗旨意识,持之以恒整治“四风”,自觉践行“三严三实”,从严整治涉企收费等行为,加强重大项目服务保障,更好地服务基层群众、服务企业发展、服务项目建设。大力发扬工匠精神,加大督查落实力度,精益求精地做细做实做好每一项工作。严格控制一般性支出和“三公”经费,继续加大民生保障领域投入,让发展成果惠及更多群众。

(四)落实主体责任,着力建设廉洁政府。认真贯彻《中国共产党廉洁自律准则》、《中国共产党纪律处分条例》和《中国共产党问责条例》,严格执行中央“八项规定”和省委“双十条”规定、市上“二十七条”实施意见,强化行政执行力,提升政府公信度。认真落实全面从严治党主体责任,加强对重点领域、关键环节、重要岗位权力运行的监督制约,强化行政监察、政务督查、效能检查和审计严查工作,严肃查处违纪违法案件,坚决防止和纠正损害群众利益的不正之风,树立清正廉洁的良好形象。

各位代表,全面建成小康社会,是新一届政府肩负的历史使命;人民对美好生活的向往,是我们履职尽责的最大动力。我们要在省委、省政府和市委的正确领导下,认真贯彻市第七次党代会精神,坚定信心、主动作为,不忘初心、继续奋进,竭尽全力打造经济繁荣发展、社会文明进步、生态舒适优美、人民富裕幸福的新天水,为与全国全省一道全面建成小康社会而努力奋斗!
